# Supplementary material for: Multi-modal machine learning approach for early detection of neurodegenerative diseases leveraging brain MRI and wearable sensor data
Source: PLOS Digit Health. 2025 Apr 25;4(4):e0000795. doi: 10.1371/journal.pdig.0000795 (PMC12027105; doi:10.1371/journal.pdig.0000795)
Supplement: S1 Table — (DOCX) [file pdig.0000795.s001.docx]

**S1 Table: T1 Brain MRI features and the related field IDs in UK Biobank**

| **Field ID** | **Description** |
| --- | --- |
| 25888 | Volume of grey matter in Amygdala (left) |
| 25889 | Volume of grey matter in Amygdala (right) |
| 25822 | Volume of grey matter in Angular Gyrus (left) |
| 25823 | Volume of grey matter in Angular Gyrus (right) |
| 25892 | Volume of grey matter in Brain-Stem |
| 25880 | Volume of grey matter in Caudate (left) |
| 25881 | Volume of grey matter in Caudate (right) |
| 25864 | Volume of grey matter in Central Opercular Cortex (left) |
| 25865 | Volume of grey matter in Central Opercular Cortex (right) |
| 25838 | Volume of grey matter in Cingulate Gyrus, anterior division (left) |
| 25839 | Volume of grey matter in Cingulate Gyrus, anterior division (right) |
| 25840 | Volume of grey matter in Cingulate Gyrus, posterior division (left) |
| 25841 | Volume of grey matter in Cingulate Gyrus, posterior division (right) |
| 25900 | Volume of grey matter in Crus I Cerebellum (left) |
| 25902 | Volume of grey matter in Crus I Cerebellum (right) |
| 25901 | Volume of grey matter in Crus I Cerebellum (vermis) |
| 25903 | Volume of grey matter in Crus II Cerebellum (left) |
| 25905 | Volume of grey matter in Crus II Cerebellum (right) |
| 25904 | Volume of grey matter in Crus II Cerebellum (vermis) |
| 25844 | Volume of grey matter in Cuneal Cortex (left) |
| 25845 | Volume of grey matter in Cuneal Cortex (right) |
| 25830 | Volume of grey matter in Frontal Medial Cortex (left) |
| 25831 | Volume of grey matter in Frontal Medial Cortex (right) |
| 25862 | Volume of grey matter in Frontal Operculum Cortex (left) |
| 25863 | Volume of grey matter in Frontal Operculum Cortex (right) |
| 25846 | Volume of grey matter in Frontal Orbital Cortex (left) |
| 25847 | Volume of grey matter in Frontal Orbital Cortex (right) |
| 25782 | Volume of grey matter in Frontal Pole (left) |
| 25783 | Volume of grey matter in Frontal Pole (right) |
| 25870 | Volume of grey matter in Heschl's Gyrus (includes H1 and H2) (left) |
| 25871 | Volume of grey matter in Heschl's Gyrus (includes H1 and H2) (right) |
| 25886 | Volume of grey matter in Hippocampus (left) |
| 25887 | Volume of grey matter in Hippocampus (right) |
| 25893 | Volume of grey matter in I-IV Cerebellum (left) |
| 25894 | Volume of grey matter in I-IV Cerebellum (right) |
| 25915 | Volume of grey matter in IX Cerebellum (left) |
| 25917 | Volume of grey matter in IX Cerebellum (right) |
| 25916 | Volume of grey matter in IX Cerebellum (vermis) |
| 25792 | Volume of grey matter in Inferior Frontal Gyrus, pars opercularis (left) |
| 25793 | Volume of grey matter in Inferior Frontal Gyrus, pars opercularis (right) |
| 25790 | Volume of grey matter in Inferior Frontal Gyrus, pars triangularis (left) |
| 25791 | Volume of grey matter in Inferior Frontal Gyrus, pars triangularis (right) |
| 25808 | Volume of grey matter in Inferior Temporal Gyrus, anterior division (left) |
| 25809 | Volume of grey matter in Inferior Temporal Gyrus, anterior division (right) |
| 25810 | Volume of grey matter in Inferior Temporal Gyrus, posterior division (left) |
| 25811 | Volume of grey matter in Inferior Temporal Gyrus, posterior division (right) |
| 25812 | Volume of grey matter in Inferior Temporal Gyrus, temporooccipital part (left) |
| 25813 | Volume of grey matter in Inferior Temporal Gyrus, temporooccipital part (right) |
| 25784 | Volume of grey matter in Insular Cortex (left) |
| 25785 | Volume of grey matter in Insular Cortex (right) |
| 25828 | Volume of grey matter in Intracalcarine Cortex (left) |
| 25829 | Volume of grey matter in Intracalcarine Cortex (right) |
| 25832 | Volume of grey matter in Juxtapositional Lobule Cortex (formerly Supplementary Motor Cortex) (left) |
| 25833 | Volume of grey matter in Juxtapositional Lobule Cortex (formerly Supplementary Motor Cortex) (right) |
| 25826 | Volume of grey matter in Lateral Occipital Cortex, inferior division (left) |
| 25827 | Volume of grey matter in Lateral Occipital Cortex, inferior division (right) |
| 25824 | Volume of grey matter in Lateral Occipital Cortex, superior division (left) |
| 25825 | Volume of grey matter in Lateral Occipital Cortex, superior division (right) |
| 25852 | Volume of grey matter in Lingual Gyrus (left) |
| 25853 | Volume of grey matter in Lingual Gyrus (right) |
| 25788 | Volume of grey matter in Middle Frontal Gyrus (left) |
| 25789 | Volume of grey matter in Middle Frontal Gyrus (right) |
| 25802 | Volume of grey matter in Middle Temporal Gyrus, anterior division (left) |
| 25803 | Volume of grey matter in Middle Temporal Gyrus, anterior division (right) |
| 25804 | Volume of grey matter in Middle Temporal Gyrus, posterior division (left) |
| 25805 | Volume of grey matter in Middle Temporal Gyrus, posterior division (right) |
| 25806 | Volume of grey matter in Middle Temporal Gyrus, temporooccipital part (left) |
| 25807 | Volume of grey matter in Middle Temporal Gyrus, temporooccipital part (right) |
| 25860 | Volume of grey matter in Occipital Fusiform Gyrus (left) |
| 25861 | Volume of grey matter in Occipital Fusiform Gyrus (right) |
| 25876 | Volume of grey matter in Occipital Pole (left) |
| 25877 | Volume of grey matter in Occipital Pole (right) |
| 25884 | Volume of grey matter in Pallidum (left) |
| 25885 | Volume of grey matter in Pallidum (right) |
| 25836 | Volume of grey matter in Paracingulate Gyrus (left) |
| 25837 | Volume of grey matter in Paracingulate Gyrus (right) |
| 25848 | Volume of grey matter in Parahippocampal Gyrus, anterior division (left) |
| 25849 | Volume of grey matter in Parahippocampal Gyrus, anterior division (right) |
| 25850 | Volume of grey matter in Parahippocampal Gyrus, posterior division (left) |
| 25851 | Volume of grey matter in Parahippocampal Gyrus, posterior division (right) |
| 25866 | Volume of grey matter in Parietal Operculum Cortex (left) |
| 25867 | Volume of grey matter in Parietal Operculum Cortex (right) |
| 25868 | Volume of grey matter in Planum Polare (left) |
| 25869 | Volume of grey matter in Planum Polare (right) |
| 25872 | Volume of grey matter in Planum Temporale (left) |
| 25873 | Volume of grey matter in Planum Temporale (right) |
| 25814 | Volume of grey matter in Postcentral Gyrus (left) |
| 25815 | Volume of grey matter in Postcentral Gyrus (right) |
| 25794 | Volume of grey matter in Precentral Gyrus (left) |
| 25795 | Volume of grey matter in Precentral Gyrus (right) |
| 25842 | Volume of grey matter in Precuneous Cortex (left) |
| 25843 | Volume of grey matter in Precuneous Cortex (right) |
| 25882 | Volume of grey matter in Putamen (left) |
| 25883 | Volume of grey matter in Putamen (right) |
| 25834 | Volume of grey matter in Subcallosal Cortex (left) |
| 25835 | Volume of grey matter in Subcallosal Cortex (right) |
| 25786 | Volume of grey matter in Superior Frontal Gyrus (left) |
| 25787 | Volume of grey matter in Superior Frontal Gyrus (right) |
| 25816 | Volume of grey matter in Superior Parietal Lobule (left) |
| 25817 | Volume of grey matter in Superior Parietal Lobule (right) |
| 25798 | Volume of grey matter in Superior Temporal Gyrus, anterior division (left) |
| 25799 | Volume of grey matter in Superior Temporal Gyrus, anterior division (right) |
| 25800 | Volume of grey matter in Superior Temporal Gyrus, posterior division (left) |
| 25801 | Volume of grey matter in Superior Temporal Gyrus, posterior division (right) |
| 25874 | Volume of grey matter in Supracalcarine Cortex (left) |
| 25875 | Volume of grey matter in Supracalcarine Cortex (right) |
| 25818 | Volume of grey matter in Supramarginal Gyrus, anterior division (left) |
| 25819 | Volume of grey matter in Supramarginal Gyrus, anterior division (right) |
| 25820 | Volume of grey matter in Supramarginal Gyrus, posterior division (left) |
| 25821 | Volume of grey matter in Supramarginal Gyrus, posterior division (right) |
| 25854 | Volume of grey matter in Temporal Fusiform Cortex, anterior division (left) |
| 25855 | Volume of grey matter in Temporal Fusiform Cortex, anterior division (right) |
| 25856 | Volume of grey matter in Temporal Fusiform Cortex, posterior division (left) |
| 25857 | Volume of grey matter in Temporal Fusiform Cortex, posterior division (right) |
| 25858 | Volume of grey matter in Temporal Occipital Fusiform Cortex (left) |
| 25859 | Volume of grey matter in Temporal Occipital Fusiform Cortex (right) |
| 25796 | Volume of grey matter in Temporal Pole (left) |
| 25797 | Volume of grey matter in Temporal Pole (right) |
| 25878 | Volume of grey matter in Thalamus (left) |
| 25879 | Volume of grey matter in Thalamus (right) |
| 25895 | Volume of grey matter in V Cerebellum (left) |
| 25896 | Volume of grey matter in V Cerebellum (right) |
| 25897 | Volume of grey matter in VI Cerebellum (left) |
| 25899 | Volume of grey matter in VI Cerebellum (right) |
| 25898 | Volume of grey matter in VI Cerebellum (vermis) |
| 25909 | Volume of grey matter in VIIIa Cerebellum (left) |
| 25911 | Volume of grey matter in VIIIa Cerebellum (right) |
| 25910 | Volume of grey matter in VIIIa Cerebellum (vermis) |
| 25912 | Volume of grey matter in VIIIb Cerebellum (left) |
| 25914 | Volume of grey matter in VIIIb Cerebellum (right) |
| 25913 | Volume of grey matter in VIIIb Cerebellum (vermis) |
| 25906 | Volume of grey matter in VIIb Cerebellum (left) |
| 25908 | Volume of grey matter in VIIb Cerebellum (right) |
| 25907 | Volume of grey matter in VIIb Cerebellum (vermis) |
| 25890 | Volume of grey matter in Ventral Striatum (left) |
| 25891 | Volume of grey matter in Ventral Striatum (right) |
| 25918 | Volume of grey matter in X Cerebellum (left) |
| 25919 | Volume of grey matter in X Cerebellum (vermis) |
| 25920 | Volume of grey matter in X Cerebellum (right) |
