# Supplementary material for: Multi-modal machine learning approach for early detection of neurodegenerative diseases leveraging brain MRI and wearable sensor data
Source: PLOS Digit Health. 2025 Apr 25;4(4):e0000795. doi: 10.1371/journal.pdig.0000795 (PMC12027105; doi:10.1371/journal.pdig.0000795)
Supplement: S2 Table — (DOCX) [file pdig.0000795.s002.docx]

**S2 Table: T2 Brain MRI features and the related field IDs in UK Biobank**

| **Field ID** | **Description** |
| --- | --- |
| 25926 | Intensity scaling for T2_FLAIR |
| 24486 | Total volume of deep white matter hyperintensities |
| 24485 | Total volume of peri-ventricular white matter hyperintensities |
| 25781 | Total volume of white matter hyperintensities (from T1 and T2_FLAIR images) |
| 25736 | Discrepancy between T2 FLAIR brain image and T1 brain image |
