# Supplementary material for: Multi-modal machine learning approach for early detection of neurodegenerative diseases leveraging brain MRI and wearable sensor data
Source: PLOS Digit Health. 2025 Apr 25;4(4):e0000795. doi: 10.1371/journal.pdig.0000795 (PMC12027105; doi:10.1371/journal.pdig.0000795)
Supplement: S3 Table — (DOCX) [file pdig.0000795.s003.docx]

**S3 Table: RPS scores and the related field IDs in UK Biobank**

| Field ID | Description |
| --- | --- |
| [26202](https://biobank.ndph.ox.ac.uk/ukb/field.cgi?id=26202) | [Standard PRS for age at menopause (AAM)](https://biobank.ndph.ox.ac.uk/ukb/field.cgi?id=26202) |
| [26204](https://biobank.ndph.ox.ac.uk/ukb/field.cgi?id=26204) | [Standard PRS for age-related macular degeneration (AMD)](https://biobank.ndph.ox.ac.uk/ukb/field.cgi?id=26204) |
| [26206](https://biobank.ndph.ox.ac.uk/ukb/field.cgi?id=26206) | [Standard PRS for alzheimer's disease (AD)](https://biobank.ndph.ox.ac.uk/ukb/field.cgi?id=26206) |
| [26210](https://biobank.ndph.ox.ac.uk/ukb/field.cgi?id=26210) | [Standard PRS for asthma (AST)](https://biobank.ndph.ox.ac.uk/ukb/field.cgi?id=26210) |
| [26212](https://biobank.ndph.ox.ac.uk/ukb/field.cgi?id=26212) | [Standard PRS for atrial fibrillation (AF)](https://biobank.ndph.ox.ac.uk/ukb/field.cgi?id=26212) |
| [26214](https://biobank.ndph.ox.ac.uk/ukb/field.cgi?id=26214) | [Standard PRS for bipolar disorder (BD)](https://biobank.ndph.ox.ac.uk/ukb/field.cgi?id=26214) |
| [26216](https://biobank.ndph.ox.ac.uk/ukb/field.cgi?id=26216) | [Standard PRS for body mass index (BMI)](https://biobank.ndph.ox.ac.uk/ukb/field.cgi?id=26216) |
| [26218](https://biobank.ndph.ox.ac.uk/ukb/field.cgi?id=26218) | [Standard PRS for bowel cancer (CRC)](https://biobank.ndph.ox.ac.uk/ukb/field.cgi?id=26218) |
| [26220](https://biobank.ndph.ox.ac.uk/ukb/field.cgi?id=26220) | [Standard PRS for breast cancer (BC)](https://biobank.ndph.ox.ac.uk/ukb/field.cgi?id=26220) |
| [26223](https://biobank.ndph.ox.ac.uk/ukb/field.cgi?id=26223) | [Standard PRS for cardiovascular disease (CVD)](https://biobank.ndph.ox.ac.uk/ukb/field.cgi?id=26223) |
| [26225](https://biobank.ndph.ox.ac.uk/ukb/field.cgi?id=26225) | [Standard PRS for coeliac disease (CED)](https://biobank.ndph.ox.ac.uk/ukb/field.cgi?id=26225) |
| [26227](https://biobank.ndph.ox.ac.uk/ukb/field.cgi?id=26227) | [Standard PRS for coronary artery disease (CAD)](https://biobank.ndph.ox.ac.uk/ukb/field.cgi?id=26227) |
| [26229](https://biobank.ndph.ox.ac.uk/ukb/field.cgi?id=26229) | [Standard PRS for crohn's disease (CD)](https://biobank.ndph.ox.ac.uk/ukb/field.cgi?id=26229) |
| [26232](https://biobank.ndph.ox.ac.uk/ukb/field.cgi?id=26232) | [Standard PRS for epithelial ovarian cancer (EOC)](https://biobank.ndph.ox.ac.uk/ukb/field.cgi?id=26232) |
| [26234](https://biobank.ndph.ox.ac.uk/ukb/field.cgi?id=26234) | [Standard PRS for estimated bone mineral density t-score (EBMDT)](https://biobank.ndph.ox.ac.uk/ukb/field.cgi?id=26234) |
| [26238](https://biobank.ndph.ox.ac.uk/ukb/field.cgi?id=26238) | [Standard PRS for glycated haemoglobin (HBA1C_DF)](https://biobank.ndph.ox.ac.uk/ukb/field.cgi?id=26238) |
| [26240](https://biobank.ndph.ox.ac.uk/ukb/field.cgi?id=26240) | [Standard PRS for height (HEIGHT)](https://biobank.ndph.ox.ac.uk/ukb/field.cgi?id=26240) |
| [26242](https://biobank.ndph.ox.ac.uk/ukb/field.cgi?id=26242) | [Standard PRS for high density lipoprotein cholesterol (HDL)](https://biobank.ndph.ox.ac.uk/ukb/field.cgi?id=26242) |
| [26244](https://biobank.ndph.ox.ac.uk/ukb/field.cgi?id=26244) | [Standard PRS for hypertension (HT)](https://biobank.ndph.ox.ac.uk/ukb/field.cgi?id=26244) |
| [26246](https://biobank.ndph.ox.ac.uk/ukb/field.cgi?id=26246) | [Standard PRS for intraocular pressure (IOP)](https://biobank.ndph.ox.ac.uk/ukb/field.cgi?id=26246) |
| [26248](https://biobank.ndph.ox.ac.uk/ukb/field.cgi?id=26248) | [Standard PRS for ischaemic stroke (ISS)](https://biobank.ndph.ox.ac.uk/ukb/field.cgi?id=26248) |
| [26250](https://biobank.ndph.ox.ac.uk/ukb/field.cgi?id=26250) | [Standard PRS for low density lipoprotein cholesterol (LDL_SF)](https://biobank.ndph.ox.ac.uk/ukb/field.cgi?id=26250) |
| [26252](https://biobank.ndph.ox.ac.uk/ukb/field.cgi?id=26252) | [Standard PRS for melanoma (MEL)](https://biobank.ndph.ox.ac.uk/ukb/field.cgi?id=26252) |
| [26254](https://biobank.ndph.ox.ac.uk/ukb/field.cgi?id=26254) | [Standard PRS for multiple sclerosis (MS)](https://biobank.ndph.ox.ac.uk/ukb/field.cgi?id=26254) |
| [26258](https://biobank.ndph.ox.ac.uk/ukb/field.cgi?id=26258) | [Standard PRS for osteoporosis (OP)](https://biobank.ndph.ox.ac.uk/ukb/field.cgi?id=26258) |
| [26260](https://biobank.ndph.ox.ac.uk/ukb/field.cgi?id=26260) | [Standard PRS for parkinson's disease (PD)](https://biobank.ndph.ox.ac.uk/ukb/field.cgi?id=26260) |
| [26265](https://biobank.ndph.ox.ac.uk/ukb/field.cgi?id=26265) | [Standard PRS for primary open angle glaucoma (POAG)](https://biobank.ndph.ox.ac.uk/ukb/field.cgi?id=26265) |
| [26267](https://biobank.ndph.ox.ac.uk/ukb/field.cgi?id=26267) | [Standard PRS for prostate cancer (PC)](https://biobank.ndph.ox.ac.uk/ukb/field.cgi?id=26267) |
| [26269](https://biobank.ndph.ox.ac.uk/ukb/field.cgi?id=26269) | [Standard PRS for psoriasis (PSO)](https://biobank.ndph.ox.ac.uk/ukb/field.cgi?id=26269) |
| [21150](https://biobank.ndph.ox.ac.uk/ukb/field.cgi?id=21150) | [Standard PRS for resting heart rate (RHR)](https://biobank.ndph.ox.ac.uk/ukb/field.cgi?id=21150) |
| [26273](https://biobank.ndph.ox.ac.uk/ukb/field.cgi?id=26273) | [Standard PRS for rheumatoid arthritis (RA)](https://biobank.ndph.ox.ac.uk/ukb/field.cgi?id=26273) |
| [26275](https://biobank.ndph.ox.ac.uk/ukb/field.cgi?id=26275) | [Standard PRS for schizophrenia (SCZ)](https://biobank.ndph.ox.ac.uk/ukb/field.cgi?id=26275) |
| [26278](https://biobank.ndph.ox.ac.uk/ukb/field.cgi?id=26278) | [Standard PRS for systemic lupus erythematosus (SLE)](https://biobank.ndph.ox.ac.uk/ukb/field.cgi?id=26278) |
| [21151](https://biobank.ndph.ox.ac.uk/ukb/field.cgi?id=21151) | [Standard PRS for total cholesterol (TCH)](https://biobank.ndph.ox.ac.uk/ukb/field.cgi?id=21151) |
| [21152](https://biobank.ndph.ox.ac.uk/ukb/field.cgi?id=21152) | [Standard PRS for total triglyceride (TTG)](https://biobank.ndph.ox.ac.uk/ukb/field.cgi?id=21152) |
| [26283](https://biobank.ndph.ox.ac.uk/ukb/field.cgi?id=26283) | [Standard PRS for type 1 diabetes (T1D)](https://biobank.ndph.ox.ac.uk/ukb/field.cgi?id=26283) |
| [26285](https://biobank.ndph.ox.ac.uk/ukb/field.cgi?id=26285) | [Standard PRS for type 2 diabetes (T2D)](https://biobank.ndph.ox.ac.uk/ukb/field.cgi?id=26285) |
| [26287](https://biobank.ndph.ox.ac.uk/ukb/field.cgi?id=26287) | [Standard PRS for ulcerative colitis (UC)](https://biobank.ndph.ox.ac.uk/ukb/field.cgi?id=26287) |
| [26289](https://biobank.ndph.ox.ac.uk/ukb/field.cgi?id=26289) | [Standard PRS for venous thromboembolic disease (VTE)](https://biobank.ndph.ox.ac.uk/ukb/field.cgi?id=26289) |
