# Supplementary material for: Multi-modal machine learning approach for early detection of neurodegenerative diseases leveraging brain MRI and wearable sensor data
Source: PLOS Digit Health. 2025 Apr 25;4(4):e0000795. doi: 10.1371/journal.pdig.0000795 (PMC12027105; doi:10.1371/journal.pdig.0000795)
Supplement: S4 Table — (DOCX) [file pdig.0000795.s004.docx]

**S4 Table: Accelerometry average data and the related field IDs in UK Biobank**

| Field ID | Description |
| --- | --- |
| [90012](https://biobank.ndph.ox.ac.uk/ukb/field.cgi?id=90012) | [Overall acceleration average](https://biobank.ndph.ox.ac.uk/ukb/field.cgi?id=90012) |
| [90013](https://biobank.ndph.ox.ac.uk/ukb/field.cgi?id=90013) | [Standard deviation of acceleration](https://biobank.ndph.ox.ac.uk/ukb/field.cgi?id=90013) |
| [90027](https://biobank.ndph.ox.ac.uk/ukb/field.cgi?id=90027) | [Average acceleration 00:00 - 00:59](https://biobank.ndph.ox.ac.uk/ukb/field.cgi?id=90027) |
| [90028](https://biobank.ndph.ox.ac.uk/ukb/field.cgi?id=90028) | [Average acceleration 01:00 - 01:59](https://biobank.ndph.ox.ac.uk/ukb/field.cgi?id=90028) |
| [90029](https://biobank.ndph.ox.ac.uk/ukb/field.cgi?id=90029) | [Average acceleration 02:00 - 02:59](https://biobank.ndph.ox.ac.uk/ukb/field.cgi?id=90029) |
| [90030](https://biobank.ndph.ox.ac.uk/ukb/field.cgi?id=90030) | [Average acceleration 03:00 - 03:59](https://biobank.ndph.ox.ac.uk/ukb/field.cgi?id=90030) |
| [90031](https://biobank.ndph.ox.ac.uk/ukb/field.cgi?id=90031) | [Average acceleration 04:00 - 04:59](https://biobank.ndph.ox.ac.uk/ukb/field.cgi?id=90031) |
| [90032](https://biobank.ndph.ox.ac.uk/ukb/field.cgi?id=90032) | [Average acceleration 05:00 - 05:59](https://biobank.ndph.ox.ac.uk/ukb/field.cgi?id=90032) |
| [90033](https://biobank.ndph.ox.ac.uk/ukb/field.cgi?id=90033) | [Average acceleration 06:00 - 06:59](https://biobank.ndph.ox.ac.uk/ukb/field.cgi?id=90033) |
| [90034](https://biobank.ndph.ox.ac.uk/ukb/field.cgi?id=90034) | [Average acceleration 07:00 - 07:59](https://biobank.ndph.ox.ac.uk/ukb/field.cgi?id=90034) |
| [90035](https://biobank.ndph.ox.ac.uk/ukb/field.cgi?id=90035) | [Average acceleration 08:00 - 08:59](https://biobank.ndph.ox.ac.uk/ukb/field.cgi?id=90035) |
| [90036](https://biobank.ndph.ox.ac.uk/ukb/field.cgi?id=90036) | [Average acceleration 09:00 - 09:59](https://biobank.ndph.ox.ac.uk/ukb/field.cgi?id=90036) |
| [90037](https://biobank.ndph.ox.ac.uk/ukb/field.cgi?id=90037) | [Average acceleration 10:00 - 10:59](https://biobank.ndph.ox.ac.uk/ukb/field.cgi?id=90037) |
| [90038](https://biobank.ndph.ox.ac.uk/ukb/field.cgi?id=90038) | [Average acceleration 11:00 - 11:59](https://biobank.ndph.ox.ac.uk/ukb/field.cgi?id=90038) |
| [90039](https://biobank.ndph.ox.ac.uk/ukb/field.cgi?id=90039) | [Average acceleration 12:00 - 12:59](https://biobank.ndph.ox.ac.uk/ukb/field.cgi?id=90039) |
| [90040](https://biobank.ndph.ox.ac.uk/ukb/field.cgi?id=90040) | [Average acceleration 13:00 - 13:59](https://biobank.ndph.ox.ac.uk/ukb/field.cgi?id=90040) |
| [90041](https://biobank.ndph.ox.ac.uk/ukb/field.cgi?id=90041) | [Average acceleration 14:00 - 14:59](https://biobank.ndph.ox.ac.uk/ukb/field.cgi?id=90041) |
| [90042](https://biobank.ndph.ox.ac.uk/ukb/field.cgi?id=90042) | [Average acceleration 15:00 - 15:59](https://biobank.ndph.ox.ac.uk/ukb/field.cgi?id=90042) |
| [90043](https://biobank.ndph.ox.ac.uk/ukb/field.cgi?id=90043) | [Average acceleration 16:00 - 16:59](https://biobank.ndph.ox.ac.uk/ukb/field.cgi?id=90043) |
| [90044](https://biobank.ndph.ox.ac.uk/ukb/field.cgi?id=90044) | [Average acceleration 17:00 - 17:59](https://biobank.ndph.ox.ac.uk/ukb/field.cgi?id=90044) |
| [90045](https://biobank.ndph.ox.ac.uk/ukb/field.cgi?id=90045) | [Average acceleration 18:00 - 18:59](https://biobank.ndph.ox.ac.uk/ukb/field.cgi?id=90045) |
| [90046](https://biobank.ndph.ox.ac.uk/ukb/field.cgi?id=90046) | [Average acceleration 19:00 - 19:59](https://biobank.ndph.ox.ac.uk/ukb/field.cgi?id=90046) |
| [90047](https://biobank.ndph.ox.ac.uk/ukb/field.cgi?id=90047) | [Average acceleration 20:00 - 20:59](https://biobank.ndph.ox.ac.uk/ukb/field.cgi?id=90047) |
| [90048](https://biobank.ndph.ox.ac.uk/ukb/field.cgi?id=90048) | [Average acceleration 21:00 - 21:59](https://biobank.ndph.ox.ac.uk/ukb/field.cgi?id=90048) |
| [90049](https://biobank.ndph.ox.ac.uk/ukb/field.cgi?id=90049) | [Average acceleration 22:00 - 22:59](https://biobank.ndph.ox.ac.uk/ukb/field.cgi?id=90049) |
| [90050](https://biobank.ndph.ox.ac.uk/ukb/field.cgi?id=90050) | [Average acceleration 23:00 - 23:59](https://biobank.ndph.ox.ac.uk/ukb/field.cgi?id=90050) |
| [90019](https://biobank.ndph.ox.ac.uk/ukb/field.cgi?id=90019) | [Monday average acceleration](https://biobank.ndph.ox.ac.uk/ukb/field.cgi?id=90019) |
| [90020](https://biobank.ndph.ox.ac.uk/ukb/field.cgi?id=90020) | [Tuesday average acceleration](https://biobank.ndph.ox.ac.uk/ukb/field.cgi?id=90020) |
| [90021](https://biobank.ndph.ox.ac.uk/ukb/field.cgi?id=90021) | [Wednesday average acceleration](https://biobank.ndph.ox.ac.uk/ukb/field.cgi?id=90021) |
| [90022](https://biobank.ndph.ox.ac.uk/ukb/field.cgi?id=90022) | [Thursday average acceleration](https://biobank.ndph.ox.ac.uk/ukb/field.cgi?id=90022) |
| [90023](https://biobank.ndph.ox.ac.uk/ukb/field.cgi?id=90023) | [Friday average acceleration](https://biobank.ndph.ox.ac.uk/ukb/field.cgi?id=90023) |
| [90024](https://biobank.ndph.ox.ac.uk/ukb/field.cgi?id=90024) | [Saturday average acceleration](https://biobank.ndph.ox.ac.uk/ukb/field.cgi?id=90024) |
| [90025](https://biobank.ndph.ox.ac.uk/ukb/field.cgi?id=90025) | [Sunday average acceleration](https://biobank.ndph.ox.ac.uk/ukb/field.cgi?id=90025) |
