# Supplementary material for: Multi-modal machine learning approach for early detection of neurodegenerative diseases leveraging brain MRI and wearable sensor data
Source: PLOS Digit Health. 2025 Apr 25;4(4):e0000795. doi: 10.1371/journal.pdig.0000795 (PMC12027105; doi:10.1371/journal.pdig.0000795)
Supplement: S5 Table — (DOCX) [file pdig.0000795.s005.docx]

**S5 Table: Accelerometry-derived data and the related field IDs in UK Biobank**

| Field ID | Description |
| --- | --- |
| [40044](https://biobank.ndph.ox.ac.uk/ukb/field.cgi?id=40044) | [Light - Day average](https://biobank.ndph.ox.ac.uk/ukb/field.cgi?id=40044) |
| [40032](https://biobank.ndph.ox.ac.uk/ukb/field.cgi?id=40032) | [Light - Day hour average](https://biobank.ndph.ox.ac.uk/ukb/field.cgi?id=40032) |
| [40048](https://biobank.ndph.ox.ac.uk/ukb/field.cgi?id=40048) | [Light - Overall average](https://biobank.ndph.ox.ac.uk/ukb/field.cgi?id=40048) |
| [40036](https://biobank.ndph.ox.ac.uk/ukb/field.cgi?id=40036) | [Light - Weekday hour average](https://biobank.ndph.ox.ac.uk/ukb/field.cgi?id=40036) |
| [40040](https://biobank.ndph.ox.ac.uk/ukb/field.cgi?id=40040) | [Light - Weekend hour average](https://biobank.ndph.ox.ac.uk/ukb/field.cgi?id=40040) |
| [40045](https://biobank.ndph.ox.ac.uk/ukb/field.cgi?id=40045) | [Moderate-Vigorous - Day average](https://biobank.ndph.ox.ac.uk/ukb/field.cgi?id=40045) |
| [40033](https://biobank.ndph.ox.ac.uk/ukb/field.cgi?id=40033) | [Moderate-Vigorous - Day hour average](https://biobank.ndph.ox.ac.uk/ukb/field.cgi?id=40033) |
| [40049](https://biobank.ndph.ox.ac.uk/ukb/field.cgi?id=40049) | [Moderate-Vigorous - Overall average](https://biobank.ndph.ox.ac.uk/ukb/field.cgi?id=40049) |
| [40037](https://biobank.ndph.ox.ac.uk/ukb/field.cgi?id=40037) | [Moderate-Vigorous - Weekday hour average](https://biobank.ndph.ox.ac.uk/ukb/field.cgi?id=40037) |
| [40041](https://biobank.ndph.ox.ac.uk/ukb/field.cgi?id=40041) | [Moderate-Vigorous - Weekend hour average](https://biobank.ndph.ox.ac.uk/ukb/field.cgi?id=40041) |
| [40043](https://biobank.ndph.ox.ac.uk/ukb/field.cgi?id=40043) | [Sedentary - Day average](https://biobank.ndph.ox.ac.uk/ukb/field.cgi?id=40043) |
| [40031](https://biobank.ndph.ox.ac.uk/ukb/field.cgi?id=40031) | [Sedentary - Day hour average](https://biobank.ndph.ox.ac.uk/ukb/field.cgi?id=40031) |
| [40047](https://biobank.ndph.ox.ac.uk/ukb/field.cgi?id=40047) | [Sedentary - Overall average](https://biobank.ndph.ox.ac.uk/ukb/field.cgi?id=40047) |
| [40035](https://biobank.ndph.ox.ac.uk/ukb/field.cgi?id=40035) | [Sedentary - Weekday hour average](https://biobank.ndph.ox.ac.uk/ukb/field.cgi?id=40035) |
| [40039](https://biobank.ndph.ox.ac.uk/ukb/field.cgi?id=40039) | [Sedentary - Weekend hour average](https://biobank.ndph.ox.ac.uk/ukb/field.cgi?id=40039) |
| [40042](https://biobank.ndph.ox.ac.uk/ukb/field.cgi?id=40042) | [Sleep - Day average](https://biobank.ndph.ox.ac.uk/ukb/field.cgi?id=40042) |
| [40030](https://biobank.ndph.ox.ac.uk/ukb/field.cgi?id=40030) | [Sleep - Day hour average](https://biobank.ndph.ox.ac.uk/ukb/field.cgi?id=40030) |
| [40046](https://biobank.ndph.ox.ac.uk/ukb/field.cgi?id=40046) | [Sleep - Overall average](https://biobank.ndph.ox.ac.uk/ukb/field.cgi?id=40046) |
| [40034](https://biobank.ndph.ox.ac.uk/ukb/field.cgi?id=40034) | [Sleep - Weekday hour average](https://biobank.ndph.ox.ac.uk/ukb/field.cgi?id=40034) |
| [40038](https://biobank.ndph.ox.ac.uk/ukb/field.cgi?id=40038) | [Sleep - Weekend hour average](https://biobank.ndph.ox.ac.uk/ukb/field.cgi?id=40038) |
