# Supplementary material for: Multi-modal machine learning approach for early detection of neurodegenerative diseases leveraging brain MRI and wearable sensor data
Source: PLOS Digit Health. 2025 Apr 25;4(4):e0000795. doi: 10.1371/journal.pdig.0000795 (PMC12027105; doi:10.1371/journal.pdig.0000795)
Supplement: S6 Table — (DOCX) [file pdig.0000795.s006.docx]

**S6 Table: Comparison with Common Models**

All models were tuned using 3 folder cross-validation to find the best hyper-parameter and tested on the testing set.

| Model Name | Testing AUC |
| --- | --- |
| LR | 0.772 |
| LDA | 0.626 |
| KNN | 0.532 |
| SVM | 0.752 |
| Gaussian | 0.817 |
