# Supplementary material for: Multi-modal machine learning approach for early detection of neurodegenerative diseases leveraging brain MRI and wearable sensor data
Source: PLOS Digit Health. 2025 Apr 25;4(4):e0000795. doi: 10.1371/journal.pdig.0000795 (PMC12027105; doi:10.1371/journal.pdig.0000795)
Supplement: Attachment — Submitted filename: PDIG-D-24-00170.pdf [file pdig.0000795.s007.pdf]

The paper titled "Multi-modal Machine Learning Approach for Early Detection of Neurodegenerative Diseases Leveraging Brain MRI and Wearable Sensor Data" represents a Multi-modal Machine Learning Approach. However, several areas require further development to enhance its scientific impact:

1. Improve the abstract by clearly outlining the specific aims, methods, key results, and conclusions. Mention specific neurodegenerative diseases being targeted.
2. Provide more detailed background on the burden of neurodegenerative diseases, including current statistics on incidence and prevalence.
3. Include a separate **Literature Review** section to include recent advances in MRI biomarkers and wearable sensor technology for neurodegenerative diseases.
4. Clearly articulate the research gap your study is addressing and how it advances current knowledge.
5. Explicitly state the hypothesis being tested in the study.
6. Specify which MRI brain parameters were selected and why. Include details on how these parameters are linked to neurodegenerative diseases.
7. In the **Classification Performance** section, explain the calculation of polygenic risk scores (PRS) and their relevance to neurodegenerative diseases.
8. Describe the lifestyle information collected and its potential impact on neurodegenerative disease progression.
9. Detail the data preprocessing steps for both MRI and accelerometry data to ensure reproducibility.
10. Justify the choice of XGBoost as the machine learning algorithm and explain any hyperparameter tuning performed. Implement at least five more machine learning algorithms and compare the performance.
11. Add a **Performance Matrices** section to discuss the evaluation metrics used (AUC, accuracy, etc.) and why they were chosen.
12. In the **Feature Importance** section provide a more detailed explanation of the feature importance analysis and its implications. Such as visualizing the performance with and without selecting important features.
13. In the **Statistical Analysis** section include visual graphs to present statistical analyses conducted to validate the findings, such as significance testing and confidence intervals.
14. Add a section **Comparison with Existing Methods** to compare your multi-modal model with other existing models in the literature to highlight its advantages.
15. Discuss the limitations of your study, including any potential biases or confounding factors.
16. Provide more concrete suggestions for future prospective studies, including specific types of neurodegenerative diseases to be investigated.
17. Include more figures and tables to increase reader understandability. Ensure all figures and tables are clear, and well-labeled.
18. The paper lacks a well-structured outline, making it difficult for readers to follow the flow of information. It needs clear, distinct sections for the Introduction, Literature Review, Methods, Dataset, Data Preprocessing, Performance Metrics, Result Analysis, Comparison with Existing Work, Discussion, and Conclusion. Each section should be clearly defined and logically organized to enhance readability and comprehension.
