# Supplementary material for: Multi-modal machine learning approach for early detection of neurodegenerative diseases leveraging brain MRI and wearable sensor data
Source: PLOS Digit Health. 2025 Apr 25;4(4):e0000795. doi: 10.1371/journal.pdig.0000795 (PMC12027105; doi:10.1371/journal.pdig.0000795)
Supplement: Attachment — Submitted filename: PDIG-D-24-00170_R1.pdf [file pdig.0000795.s009.pdf]

After careful review, there is still significant room for improvement in the manuscript titled "Multi-modal Machine Learning Approach for Early Detection of Neurodegenerative Diseases Leveraging Brain MRI and Wearable Sensor Data." Addressing the following areas could enhance the paper's scientific impact:

1. The abstract still lacks specificity in outlining the aims, methods, key results, and conclusions, particularly regarding the neurodegenerative diseases targeted.
2. The introduction does not provide sufficient background on the burden of neurodegenerative diseases, including critical statistics on incidence and prevalence.
3. A dedicated Literature Review section is necessary to contextualize the study within recent advances in MRI biomarkers and wearable sensor technology.
4. The research gap and hypothesis are not clearly articulated, which weakens the study's contribution to the field.
5. The selected MRI parameters and their relevance are described in insufficient detail, and polygenic risk scores and data preprocessing steps are inadequately explained.
6. The comparison with additional machine learning algorithms lacks depth and justification.
7. The manuscript lacks a conclusive section that summarizes the findings and their implications, which is essential for any scientific paper.
8. The overall manuscript structure remains unclear, making it challenging to follow the flow of information.

Addressing these points could significantly enhance the clarity and scientific impact of the manuscript.
