# Supplementary material for: Multi-modal machine learning approach for early detection of neurodegenerative diseases leveraging brain MRI and wearable sensor data
Source: PLOS Digit Health. 2025 Apr 25;4(4):e0000795. doi: 10.1371/journal.pdig.0000795 (PMC12027105; doi:10.1371/journal.pdig.0000795)
Supplement: Attachment — Submitted filename: Response to Reviewers_updated.docx [file pdig.0000795.s008.docx]

**PLOS Digital Health Rebuttal**

Reviewer #1: The paper titled "Multi-modal Machine Learning Approach for Early Detection of Neurodegenerative Diseases Leveraging Brain MRI and Wearable Sensor Data" represents a Multi-modal Machine Learning Approach. However, several areas require further development to enhance its scientific impact:

1.	Improve the abstract by clearly outlining the specific aims, methods, key results, and conclusions. Mention specific neurodegenerative diseases being targeted.

Thank you for your suggestions. We have improved and re-organised the abstract based on these comments.

2.	Provide more detailed background on the burden of neurodegenerative diseases, including current statistics on incidence and prevalence.

Thank you for your suggestion. We have included a detailed background on the burden in the introduction section (lines 80-91).

3.	Include a separate Literature Review section to include recent advances in MRI biomarkers and wearable sensor technology for neurodegenerative diseases.

Thank you for your suggestion. We have included two detailed subsections in the introduction to address the recent advances of these two modalities.

4.	Clearly articulate the research gap your study is addressing and how it advances current knowledge.

Thank you for your comments. We have included a “Study Objective” subsection at the end of the introduction to clearly articulate the research gap and the goal of our study.

5.	Explicitly state the hypothesis being tested in the study.

We have stated our hypothesis in the “Study Objective” subsection in the introduction.

6.	Specify which MRI brain parameters were selected and why. Include details on how these parameters are linked to neurodegenerative diseases.

Thank you for your suggestions. We have listed the detailed MRI brain parameters as supplementary 1 and 2 and added details about why those features were chosen in the Method section, from line 398 to line 407.

7.	In the Classification Performance section, explain the calculation of polygenic risk scores (PRS) and their relevance to neurodegenerative diseases.

Thank you for your suggestions. We have added a new sub-section under Method section to explain the PRS’s information source and their relevance to neurodegenerative diseases. You can find the detailed information from line 415 to line 420 and Supplementary 3.

8.	Describe the lifestyle information collected and its potential impact on neurodegenerative disease progression.

Thank you for your suggestions. We have added a new sub-section under Method section to explain the lifestyle data source and their relevance to neurodegenerative diseases. You can find the detailed information from line 434 to line 439.

9.	Detail the data preprocessing steps for both MRI and accelerometry data to ensure reproducibility.

We have included a supplementary section within the Method section to provide a detailed explanation of the accelerometry data that was utilised. The comprehensive information can be located between lines 425 and 4.

The data pretreatment methods for both MRI and accelerometry data were performed by UK Biobank, ensuring reproducibility and eliminating any concerns regarding data quality. As these are pre-processed by the UKB, the same data can be used by subsequent researchers to ensure quality and reproducibility.

10.	Justify the choice of XGBoost as the machine learning algorithm and explain any hyperparameter tuning performed. Implement at least five more machine learning algorithms and compare the performance.

Thank you for your suggestions. We have added a new paragraph to justify why we apply XGBoost model and added the hyperparameter detailed.

As for ML algorithms, we have a supplementary section to compare the XGBoost model with 5 traditional ML models.

11.	Add a Performance Matrices section to discuss the evaluation metrics used (AUC, accuracy, etc.) and why they were chosen.

Thank you for your suggestions. We have added a paragraph under the “Classification Performance” Section, discussing the evaluation metrics used and why they were chosen (lines 459-473).

12.	In the Feature Importance section provide a more detailed explanation of the feature importance analysis and its implications. Such as visualizing the performance with and without selecting important features.

Thank you for your suggestions. We have added a new paragraph under “Feature Importance” Section, to illustrate the feature importance analysis algorithm and its implications. You can find the detailed changes from line 252 to 257.

13.	In the Statistical Analysis section include visual graphs to present statistical analyses conducted to validate the findings, such as significance testing and confidence intervals.

Thank you for your suggestions. We have added significance testing in Table 1.

14.	Add a section Comparison with Existing Methods to compare your multi-modal model with other existing models in the literature to highlight its advantages.

Thank you for your suggestions. We have compared the XGBoost model with 5 traditional ML models as supplementary X.

15.	Discuss the limitations of your study, including any potential biases or confounding factors.

Thank you for your comments. We have included the limitations of our study including potential bias (see lines 343-355).

16.	Provide more concrete suggestions for future prospective studies, including specific types of neurodegenerative diseases to be investigated.

Thank you for your comments. We have included the limitations of our study including potential bias (see lines 343-355).

17.	Include more figures and tables to increase reader understandability. Ensure all figures and tables are clear, and well-labeled.

Thank you for your comments. We have revised some figures and tables as suggested.

18.	The paper lacks a well-structured outline, making it difficult for readers to follow the flow of information. It needs clear, distinct sections for the Introduction, Literature Review, Methods, Dataset, Data Preprocessing, Performance Metrics, Result Analysis, Comparison with Existing Work, Discussion, and Conclusion. Each section should be clearly defined and logically organized to enhance readability and comprehension.

By addressing these comments, the paper can be significantly improved in terms of scientific precision, clarity, and overall impact on the field of neurodegenerative disease research.

Thank you for your comments. We have extensively revised and made several added subsections to the manuscript to aid readability. The overall sections are adhered to the guidance as per journal requirements.

Reviewer #2: The primary goal of this study is to investigate the combination of accelerometry data from wearable devices and MRI-based brain parameters in predicting the incidence of neurodegenerative diseases. However, the authors should concentrate on the following issues before resubmission:

(i) The authors should show the contributions in point-by-point basis.

The rebuttal are done as per point by point basis as requested.

(ii) The authors should add a literature review section as well.

The introduction and discussion section has been comprehensively revised with added literature review covering the topic.

(iii) The authors should elaborate the technical method applied here in the study.

This has been expanded in the Methods section of the revised manuscript.

(iv) The authors should also show the results of more analysis to come to the conclusion.

Thank you for the comments. We have clarified our results section and revised the discussion section to discuss more regarding our salient findings.

Reviewer #3: Suggestions for Improvement formatting

Consolidate Related Questions:

Group similar questions together to avoid redundancy.

Use Numbering or Bullet Points:

Organize questions with clear numbering or bullet points.

Clear Headers or Titles:

Provide headers for different sections of questions to enhance readability.

Structured Responses:

Respond to each question in a structured format, corresponding to the numbering or bullet points used for questions.

Seek Clarification if Needed:

Request clarification on unclear or repetitive questions to streamline communication.

Thank you for your suggestions. We have tried to enhance and amendment our manuscript but within the confined of the journal guidelines.

Novelty

What is the primary focus of the study regarding neurodegenerative diseases?

It is to investigate if the combination of MRI brain parameters and activity accelerometery data can be used to predict the incidence of neurodegenerative diseases. We have stated this in the study objectives.

How many participants were involved in the study cohort, and where were they sourced from?

A total of 19793 participants from the UK Biobank Study.

What types of data were collected from the participants?

Brain MRI (T1 and T2 W sequences) and Accelerometry were the main data used.

We also used dempgraphics and lifestyle factors (e.g. smoking habit, alcohol consumption, duration of daytime napping, and duration of daytime dozing). These are documented in the Methods section.

How was the incidence of neurodegenerative diseases defined and recorded during the study?

Cases were defined as individuals diagnosed with any of these diseases at least one year after their image screening. The mean follow-up time for our cohort is 5.9 years. This process identified 56 positive cases, comprising 19 with PD, 13 with AD, and 40 with dementia. We have documented this in the Results section. These outcomes were algorithmically derived from the UK Biobank organisation using linked

Which machine learning algorithm was used, and what was its purpose in the study?

We used XGBoost (Extreme Gradient Boosting) which is increasingly utilized in medical classification tasks due to its robust performance and interpretability. It consistently outperforms traditional models like logistic regression in various medical studies. We have included explanation in the expanded Methods section.

What was the highest achieved AUC value, and which model configuration achieved it?

The highest AUC was from using all modalities (model 1) with AUC of 0.819. Please refer to Table 2.

According to the feature importance analysis, what were the top contributors to predicting neurodegenerative disease incidence?

Please refer to Figure 2 for the top 20 features of feature important analysis. The higher the number, the more important the features.

Major concern

What specific empirical evidence or studies demonstrate a clear improvement in predictive accuracy when combining accelerometry data and MRI-based brain parameters compared to using either method alone?

When we performed the so called ‘ablation studies’, the model performance reduced.

Notably, brain MRI data contributed the most to the final prediction, with its removal resulting in a significant decrease of 0.131 in AUC compared to Model 1. Conversely, the exclusion of PRS and lifestyle information led to relatively smaller reductions in AUC, decreasing by 0.06 and 0.061, respectively.

How do these studies control for potential confounding variables and biases that may influence the observed outcomes?

We have tried to mitigate the impact of lifestyle factors, by incorporating features such as smoking status, drinking status and frequencies, nap time during day, and daytime dozing/sleeping and lifestyle factors, into our model. Although some biases from other factors may exist, it was not possible to account for all potential confounders based on the available data.

Limitations of MRI Parameters:

Given the heterogeneous nature of neurodegenerative diseases, what are the acknowledged limitations of MRI-based parameters in accurately capturing disease progression and early pathological changes?

Qualitative and quantitative assessment of MRI brain parameters, such as atrophy patterns, white matter hyperintensities and cortical thickness, may be affected by differences in scanner specifications, settings and parameters, which can result in failure to detect disease progression or early changes. Also, detection of these parameters may sometimes only be feasible with serial imaging, not just from imaging at one specific timepoint. Most importantly, patients with neurodegenerative diseases often have co-morbidities or multiple types of neurodegenerative diseases, causing difficulty in imaging interpretation and thus diagnosis.

How do these limitations impact the reliability and generalizability of using MRI data as a biomarker for predicting disease incidence?

Though certain MRI parameters have been confirmed to be reliable biomarkers, such as hippocampal atrophy for Alzheimer’s diseases, others such as white matter hyperintensities, can be identified in a multitude of neurodegenerative diseases, as well as other pathologies, such as stroke. Hence, not all previously evidence-supported imaging biomarkers may be useful surrogates for neurodegenerative disease incidence as a whole.

Accuracy and Reliability of Accelerometry Data:

What methodologies or validation studies support the accuracy and reliability of accelerometry data from wearable devices in capturing subtle movement patterns and motor abnormalities associated with neurodegenerative diseases?

Thank you for your comment. We have included details of these methodologies and studies in the introduction section (lines 175-188).

How do factors such as device variability, data processing algorithms, and participant compliance affect the consistency and interpretability of accelerometry data?

Thank you for your comment; this is an important question. In this study, we utilized preprocessed data from the UK Biobank, which enhances the consistency of results since all researchers will be using the same standardized dataset.

1) To address potential biases caused by device variability, we applied standard normalization and processing methods. These methods help ensure that differences in device sensitivity and calibration do not affect the data interpretation, allowing for more reliable comparisons across participants.
2) We also considered participant compliance, as variations in wear time can impact data quality. By using a standardized approach to data processing, we aimed to minimize the effects of non-compliance on the overall dataset.
3) Furthermore, we employed the XGBoost model for our analysis. This model is particularly effective in handling complex data structures and can account for interactions between variables, which helps to mitigate the influence of potential biases in the data.

Comparison with Alternative Predictors:

In comparative terms, how does the predictive performance of combining accelerometry and MRI data stack up against alternative predictors like genetic markers, lifestyle factors, or other advanced imaging techniques (e.g., PET scans, fMRI)?

Thank you for your comments and raising this is important question.

In our study, the combination of accelerometry and MRI data achieved an Area Under the Curve (AUC) of 0.741, indicating a moderate level of predictive performance. For a detailed comparison with other alternative predictors, including genetic markers and lifestyle factors, please refer to Table 2, which outlines the performance metrics for each predictor.

Unfortunately, we do not currently have access to data from other advanced imaging techniques, such as PET scans or fMRI. We recognize that these modalities could provide valuable insights and enhance predictive accuracy. We plan to explore the integration of such data in future research once it becomes available.

What are the strengths and weaknesses of each predictor in terms of sensitivity, specificity, and clinical applicability?

We appreciate your valuable comments and suggestion. Whilst we have not done sensitivity and specificity analysis for each predictor, it would be useful to perform for future. However, as each individual MRI or accelerometry data are combined, it is more useful to view them in combination as shown, rather than individually, as rarely we will use one or two single predictor.

Generalizability Across Neurodegenerative Diseases:

To what extent can findings from studies primarily focused on Parkinson's Disease, as highlighted by Rastegari et al. and Schalkamp et al., be extrapolated to other neurodegenerative diseases such as Alzheimer's Disease and various types of dementia?

Thank you for your comments. The machine learning techniques utilized by the above-mentioned researchers to detect subtle gait and motor abnormalities within accelerometry data can also be adapted to analyse data collected from other neurodegenerative diseases. As detailed in our revised introduction, other neurodegenerative diseases, including Alzheimer’s disease, also cause patients to exhibit gait abnormalities that can be detected on accelerometry. A recent study performed on the UK Biobank cohort showed accelerometer-derived measures to be predictive of both Alzheimer’s and Parkinson’s disease incidence (Winer et al., 2024). Of course, we agree that the majority of previous studies have focused on the utility of accelerometry data on Parkinson’s disease, rather than other neurodegenerative diseases, and recognise this as a limitation.

What disease-specific factors might influence the utility and predictive power of combined accelerometry and MRI data across different neurodegenerative conditions?

All neurodegenerative diseases, including Alzheimer’s disease, Parkinson’s disease and frontotemporal dementia, can cause patients to present with both cognitive and motor deficits. Accelerometry data from wearable devices has been demonstrated to be useful in picking up subtle gait and motor abnormalities in early stage of neurodegenerative diseases (as outlined in our revised introduction section). Combining it with established MRI brain parameters, thus, carries the potential to facilitate and improve early detection of neurodegenerative diseases.

Longitudinal Study Design:

What are the key methodological considerations and challenges associated with using longitudinal data, such as that derived from large cohorts like the UK Biobank, to investigate the predictive potential of integrated accelerometry and MRI data?

Thank you for your valuable comments. There are several, which we will briefly elaborate on here and in the Discussion. As a more general challenge, accounting for population heterogeneity in a predictive model is a potential issue. The UKB, despite its large size, is a relatively homogenous cohort consisting of British Caucasians.  In addition, integrating accelerometry and MRI data requires expertise from multiple disciplines. Future prospective studies may be able to address some of these issues.

How do study designs account for participant attrition, data heterogeneity over time, and changes in disease status to ensure robustness and reliability of findings?

Data heterogeneity within the study cohort is thought to not be a major issue, as the UKB data collection is under strict guidance. However, when findings are to be generalisable to other centres or regions, data heterogeneity could become an issue, which need to be looked at. Similarly, the change in disease status and accuracy in which these data are collected are integral to the accuracy and reproducibility of subsequent findings.

Clinical Utility and Implementation:

Beyond research settings, how might the integration of accelerometry data with MRI parameters influence clinical decision-making and patient management in terms of early detection, treatment planning, and monitoring of neurodegenerative diseases?

The potential impact in the clinical setting may be that MRI Brain will serve as one of most reliable tools in predicting and detecting early stages of neurodegenerative disease, which may result in earlier intervention for at-risk patients. Accelerometry data may serve as an adjunct predictive tool, especially in patients with subtle motor or gait abnormalities. However, the actual implementation of MRI and accelerometry as predictive tools in the clinical setting is beyond the scope of this research.

What are the potential barriers and facilitators to translating research findings into practical clinical applications, and how can these be addressed to optimize patient outcomes?

Thank you for your comments. This is an important question. The main barrier for clinical translation of our research findings is the acceptability of the predictive tool by both doctors and patients. MRI brain has been utilised by clinicians for decades as a diagnostic tool and has high acceptability. Wearable devices / accelerometers are an increasingly accepted monitoring tool in the clinical setting, though its clinical utility relies heavily on patient’s compliance. In addition, its clinical use in early detection of diseases, in general, is limited. Further clinical studies on the use of wearable devices / accelerometers not only for disease monitoring, but also disease detection, is needed to lend evidence for its effectiveness, which can help improve its acceptability. Patient education is also key to ensure these devices reach its maximal clinical utility.

These detailed questions aim to critically assess the validity, reliability, and practical implications of the hypothesis, prompting a deeper exploration of the methodological, clinical, and translational aspects surrounding the integration of accelerometry and MRI data in neurodegenerative disease prediction.

How do these studies control for potential confounding variables and biases that may influence the observed outcomes?

Thank you for your comments. We would like to emphasize that this study is a prediction study, with the primary focus on the accuracy of forecasting outcomes. While concerns about bias are generally less critical in prediction studies compared to association studies, we recognize that potential confounding variables can still influence the accuracy of predictions.

In our analysis, we utilized the XGBoost model, which is a powerful machine learning algorithm capable of capturing complex interactions and relationships within the data. This non-linear approach allows the model to account for various factors, thereby reducing the impact of potential confounders on the predictions.

Although the primary aim of our study is not to establish causal relationships, we have taken measures to minimize biases in our data collection and processing. By ensuring high-quality data preprocessing and employing robust validation techniques, we aim to enhance the reliability of our predictions.

Ethical Concern Raised:

The demographic characteristics of the study cohort raise ethical concerns regarding potential biases and implications for the generalizability of findings.

Yes, we agree with this comment. We have added this to the limitation.

Detailed Ethical Questions Raised:

Age Discrepancy Impact: How might the significant age difference between the neurodegenerative disease group and the healthy group influence the study's conclusions regarding disease prediction and risk factors?

Age is known to be a risk factor so inevitably this will play a role. The UKB study cohort is limited to participants aged 40-69 years.

Gender Disparity: What ethical considerations should be taken into account regarding the higher proportion of men in the neurodegenerative disease group compared to the healthy group? How might this gender disparity affect the interpretation of study outcomes?

Thank you for your comment. We agree that gender may play a role here, but the numbers are small, and the differences may be overinflated. For example, male with disease n=34 vs female with disease n=22. A larger cohort may be needed to discern if these differences are significant in terms of prediction.

Representation and Generalizability: To what extent do the demographic characteristics of the study cohort (e.g., age distribution, gender ratio) affect the generalizability of findings to broader populations, particularly those with different demographic profiles?

This is a good question. For this part, we acknowledge that this is a limitation of the current study in that our findings need to be tested and validated across broader population to discern generalisability.

Potential Bias in Lifestyle Factors: How might the observed differences in lifestyle factors such as smoking and drinking habits between the disease and healthy groups introduce biases into the study outcomes? What measures were taken to mitigate these biases?

Thank you for your insightful comments; this is a good question. It is important to clarify that this study is a prediction study rather than an association study. In prediction studies, the primary focus is on the accuracy of forecasting outcomes, which makes concerns about bias not critical. They would be critical in an association study that seeks to establish causal relationships.

Furthermore, in the feature importance section, we observe that lifestyle factors do not rank among the top 20 features, indicating that they have a minimal impact on the final prediction model. This suggests that other factors may play a more significant role in predicting outcomes. While we acknowledge that lifestyle factors can influence health, their limited contribution in this context reinforces the robustness of our predictive model.

Ethical Recruitment and Consent: What ethical considerations were addressed during participant recruitment and consent processes to ensure transparency, fairness, and voluntary participation, especially given the sensitive nature of neurodegenerative disease research?

The study cohort was part of the wider UK Biobank study in which recruited generally healthy individuals. Being based solely in the UK, there is an inherent selection bias towards this population. The recruitment procedure did not seek out a particular group of patients, with only hard criteria being age between 40-69 years old. However, we cannot rule out inadvertent selection bias as participation was entirely voluntary.

Equity in Access to Healthcare: Considering the study's findings may impact healthcare policies and resource allocation, what ethical obligations exist to ensure equitable access to early detection and intervention strategies identified through research?

Our research is not prospective or interventional in nature, so this cannot be directly answered, but we agree that this is an important issue.

Privacy and Confidentiality: How were participant privacy and confidentiality protected throughout the study, particularly concerning the sensitive health data collected and analyzed?

Patient privacy was key in the recruitment of UK Biobank cohort and subject identity was kept strictly confidential.

The results from Table 2 starkly expose the pivotal importance of brain MRI data in achieving high predictive accuracy, rendering the exclusion of such data in Model 2 a catastrophic misstep that significantly compromised the model's performance.

Yes, this is the idea of the ablation study to prove precisely this point.

Questions Raised:

Justification for Exclusions: What rationale guided the decision to exclude brain MRI data in Model 2, despite its demonstrated pivotal role in enhancing predictive accuracy compared to other modalities?

This is the idea of an ablation study to demonstrate the important role of brain MRI.

Impact of Lifestyle Information: How do the findings from Model 4, which omitted lifestyle information, underscore the critical role of non-genetic factors in neurodegenerative disease prediction, despite the moderate decrease in AUC compared to Model 1?

Thank you for your comments, this is an important question. When we compare the AUC values of Model 3 (0.758) and Model 4 (0.759), we observe that the lifestyle information is indeed critical, as it demonstrates that lifestyle factors contribute almost the same results as PRS to neurodegenerative disease prediction.

Then, we further excluded polygenic risk scores (PRS) from Model 3, which resulted in an AUC of 0.741. This suggested that when comparing the predictive power of lifestyle factors to that of MRI and accelerometry data, the latter appears to have a more substantial impact on prediction accuracy.

Ethical Implications: What ethical considerations arise from the decision to exclude potentially vital information sources (such as brain MRI data) from predictive models, given their substantial impact on diagnostic accuracy and patient outcomes?

This is to demonstrate the different performance between different modalities and was the reason for demonstration to show the differences that can exist.

Future Research Directions: In light of these findings, what future research avenues could explore integrating additional modalities or refining existing models to optimize predictive accuracy while maintaining ethical standards and practical feasibility?

Currently, there are very few established biomarkers for predicting neurodegenerative disease. We made some exploration of using genomics (in the form of PRS score) in this study, but further genetics related features could be explored. We have included some elaboration in the Discussion section.

Clinical Translation: How might the findings influence clinical decision-making regarding the adoption of multi-modal predictive models for neurodegenerative diseases, particularly in terms of resource allocation and patient management strategies?

The potential implication may be that MRI features will be very helpful in predicting neurodegenerative disease. However, cost-effective analysis would need to be performed to discern if more resource allocations are needed. This is an interesting point, but beyond the remit of our current study.

**Bias and Interpretation: What steps were taken to mitigate potential biases in interpreting the contribution of each modality to predictive performance, considering the complex interplay between MRI data, genetic predisposition (PRS), lifestyle factors, and accelerometry data?**

Thank you for your comments. This is an issue we have chosen to address by performing ablation studies. This means that we build separate prediction models excluding one of feature (i.e. one model excluding only brain mri features, one model only excluding accelerometry data, etc). By doing this, we can potentially discern the relative contribution, and whether synergistically, they also contribute to the prediction performance.

Transparency and Reporting: How were the results communicated transparently to stakeholders, including patients, healthcare providers, and policymakers, to ensure informed decision-making regarding the adoption of predictive models in clinical practice?

Publication of such results would be an effective way to communicate findings to the relevant stakeholders. Future prospective studies will need to be performed to validate if predictive models can be effective in real world clinical practice.

How might focusing on specific anatomical brain regions as predictive biomarkers overlook the multifaceted nature of neurodegenerative diseases, including genetic, environmental, and socio-economic factors?

We have tried to mitigate this by including lifestyle factors and PRS score, but we agree that there may be other potential interactions that we have not explored. This study give credence for future exploration in such thinking.

What ethical considerations arise from attributing predictive value to certain brain regions, particularly in terms of potential stigmatization and psychosocial impact on individuals with or at risk of neurodegenerative diseases?

Thank you for your comments. We do not foresee any immediate risk of stigmatization and psychosocial impacts at the individual basis. These biomarkers are probability based and in that sense is like many clinical biomarkers used for disease prediction.

To what extent do the identified brain regions as predictive biomarkers represent the demographic diversity of the study cohort, and how might this impact the reliability and applicability of predictive models across different populations?

Owing to large size of the sampled data, we do feel that there is demographics diversity, but given the small numbers of incident cases, there is always a risk of sampling bias. Future prospective studies across different populations will be helpful in the future.

How were participants informed about the potential implications of identifying specific brain regions as predictive biomarkers, and what measures were taken to provide adequate counseling and support?

The results are not published. In any case, the identify of participants are not identifiable owing to study design.

What ethical guidelines should be implemented to ensure responsible communication and interpretation of research findings related to specific brain regions as biomarkers for neurodegenerative diseases?

Through publication of studies as these, we hope to be effective communicator of the findings along with news articles that may better inform the general public in a more understandable format.

Reviewer #4:

1. In the abstract It is recommended to include major contributions, pitfalls of the work, and a summary of results in the paper. Not properly mentioned.

Thank you for your comments. We have made necessary changes to the abstract.

2. The introduction to the paper is poorly written. There are no challenges found in the existing works, and there is no mention of the need for this work in the current scenarios. The authors do not provide any motivation behind this article. It is recommended to provide motivation through an illustrative example for better understanding. Considering the most recent work in the literature and specifying the limitations is advised. It is recommended that the summary includes the limitations of the existing works and which limitations are addressed by the authors.

Thank you for your comments. We have made substantial changes to the introduction section to address these concerns.

3. After introduction section, direct results mentioned. Why? Their is not any flow in the manuscript.

Thank you for your comments. This is the recommended format from the journal.

4. The Results are incomplete. Mentioned in statistical analysis devised 4 models? Where are those in brief? No Results further.

Thank you for your comment. The 4 models are listed at the “Classification Performance" section, and the results are listed at Table 2.
