# Supplementary material for: Multi-modal machine learning approach for early detection of neurodegenerative diseases leveraging brain MRI and wearable sensor data
Source: PLOS Digit Health. 2025 Apr 25;4(4):e0000795. doi: 10.1371/journal.pdig.0000795 (PMC12027105; doi:10.1371/journal.pdig.0000795)
Supplement: Attachment — Submitted filename: Response to Reviewers_updated R2.docx [file pdig.0000795.s010.docx]

**PLOS Digital Health Rebuttal**

After careful review, there is still significant room for improvement in the manuscript titled "Multi-modal

Machine Learning Approach for Early Detection of Neurodegenerative Diseases Leveraging Brain MRI

and Wearable Sensor Data." Addressing the following areas could enhance the paper's scientific impact:

1. The abstract still lacks specificity in outlining the aims, methods, key results, and conclusions,

particularly regarding the neurodegenerative diseases targeted.

Author’s Reply: We have revised the abstract to clearly outline the aims, methods, key results, and conclusions, specifically focusing on the neurodegenerative diseases targeted in our study.

2. The introduction does not provide sufficient background on the burden of neurodegenerative

diseases, including critical statistics on incidence and prevalence.

Author’s Reply: The critical statistics on the incidence and prevalence of neurodegenerative diseases were outlined as follows:

`The incidence of neurodegenerative disorders in 2019 was 7,236.38 in thousands, while the prevalence was found to be 51,624.19 in thousands, both showing over 240% increase compared with data from 1990`

3. A dedicated Literature Review section is necessary to contextualize the study within recent

advances in MRI biomarkers and wearable sensor technology.

Author’s Reply: Thank you for your comments. A dedicated Literature Review section has been included previously and we do not feel that any more can be added as this will lengthen the article unnecessarily. In the said sections of “Structural MRI Brain Parameters as Imaging Biomarkers for Neurogenerative Diseases” and “Accelerometry Data from Wearable Devices as a Diagnostic and Prognostic Tool for Neurodegenerative Diseases”, we have contextualised our study within recent advances in MRI biomarkers and wearable sensor technology, highlighting the novelty and relevance of our approach.

4. The research gap and hypothesis are not clearly articulated, which weakens the study's contribution to the field.

Author’s Reply: The research gap and hypothesis have been explicitly articulated under the section “Study Objective”.

5. The selected MRI parameters and their relevance are described in insufficient detail, and polygenic

risk scores and data preprocessing steps are inadequately explained.

Author’s Reply: We have elaborated on the selected MRI parameters and their relevance, as well as provided detailed explanations of polygenic risk scores and the data preprocessing steps undertaken. Please refer to the “Methods” section under “Brain MRI Pre-Processing” and “Polygenic Risk Score Data”. Moreover, in the Discussion, we have discussed in depth the value of MRI parameters linking to evidence in the prior literature.

6. The comparison with additional machine learning algorithms lacks depth and justification.

Author’s Reply: The comparison with additional machine learning algorithms has been expanded with greater depth and justification, including a discussion of their performance and relevance to our study. We have amended it as follows:

“XGBoost, an advanced machine learning algorithm, has shown promising results in medical classification tasks. It outperforms traditional methods like logistic regression in predicting myocardial infarction, achieving higher ROC scores (Moore & Bell, 2022). XGBoost demonstrates robustness in handling missing data, maintaining high F1-scores even with 90% missing values in breast cancer and heart failure datasets (Stokanović et al., 2024). The algorithm excels at managing complex, high-dimensional data, as evidenced by its superior performance in cancer stage prediction using multi-omics data (Ma et al., 2020). Importantly, XGBoost offers interpretability through feature importance ranking and SHAP values, bridging the gap between medicine and data science (Moore & Bell, 2022; Price et al., 2022). Its ability to handle data complexities and provide interpretable results makes XGBoost a valuable tool for medical practitioners in diagnosis and treatment decision-making (Price et al., 2022).”

7. The manuscript lacks a conclusive section that summarizes the findings and their implications,

which is essential for any scientific paper.

Author’s Reply: A conclusive section summarizing the findings and their implications has been added, providing a clear and concise end to the manuscript. We have added:

“In conclusion, this study provides evidence for the strength of combining MRI brain parameters and accelerometry data from wearable devices to predict the incidence of neurodegenerative diseases. Using a cohort of 19,793 participants from the UK Biobank, free of neurodegenerative disease at baseline and followed for an average of 5.9 years, we employed the XGBoost machine learning algorithm to develop prediction models. The comprehensive model, incorporating MRI parameters, accelerometry data, polygenic risk scores, and lifestyle information, achieved the highest AUC value of 0.819. In contrast, the model excluding MRI parameters had the lowest AUC value of 0.688. Feature importance analysis highlighted that 18 of the top 20 predictors were MRI brain parameters, while 2 were from accelerometry data, emphasising the critical role of MRI parameters in predicting neurodegenerative diseases.”

8. The overall manuscript structure remains unclear, making it challenging to follow the flow of

information.

Author’s Reply: We believe these revisions significantly enhance the scientific impact and clarity of our manuscript. We are grateful for the opportunity to improve our work and look forward to your further feedback.

Thank you for your consideration.
